# Variability of structurally constrained and unconstrained functional connectivity in schizophrenia

**DOI:** 10.1002/hbm.22932

**Published:** 2015-08-14

**Authors:** Ye Yao, Lena Palaniyappan, Peter Liddle, Jie Zhang, Susan Francis, Jianfeng Feng

**Affiliations:** ^1^ Centre for Computational Systems Biology Fudan University Shanghai People's Republic of China; ^2^ School of Mathematical Sciences Fudan University Shanghai People's Republic of China; ^3^ Department of Computer Science University of Warwick Coventry United Kingdom; ^4^ Translational Neuroimaging in Mental Health, Division of Psychiatry & Applied Psychology Institute of Mental Health Nottingham United Kingdom; ^5^ Early Intervention in Psychosis, Nottinghamshire Healthcare NHS Foundation Trust Nottingham United Kingdom; ^6^ Department of Medical Imaging Jinling Hospital, Nanjing University School of Medicine Nanjing People's Republic of China; ^7^ Sir Peter Mansfield Imaging Centre, School of Physics and Astronomy University of Nottingham United Kingdom; ^8^ Shanghai Center for Mathematical Sciences, Fudan University Shanghai People's Republic of China; ^9^ School of Life Sciences and Collaborative Innovation Center for Brain Science Fudan University Shanghai People's Republic of China

**Keywords:** Schizophrenia, functional MRI, diffusion tensor imaging, functional connectivity, structurally constrained, structurally unconstrained, functional connectivity entropy

## Abstract

Spatial variation in connectivity is an integral aspect of the brain's architecture. In the absence of this variability, the brain may act as a single homogenous entity without regional specialization. In this study, we investigate the variability in functional links categorized on the basis of the presence of direct structural paths (primary) or indirect paths mediated by one (secondary) or more (tertiary) brain regions ascertained by diffusion tensor imaging. We quantified the variability in functional connectivity using an unbiased estimate of unpredictability (functional connectivity entropy) in a neuropsychiatric disorder where structure‐function relationship is considered to be abnormal; 34 patients with schizophrenia and 32 healthy controls underwent DTI and resting state functional MRI scans. Less than one‐third (27.4% in patients, 27.85% in controls) of functional links between brain regions were regarded as direct primary links on the basis of DTI tractography, while the rest were secondary or tertiary. The most significant changes in the distribution of functional connectivity in schizophrenia occur in indirect tertiary paths with no direct axonal linkage in both early (*P* = 0.0002, *d* = 1.46) and late (*P* = 1 × 10^−17^, *d* = 4.66) stages of schizophrenia, and are not altered by the severity of symptoms, suggesting that this is an invariant feature of this illness. Unlike those with early stage illness, patients with chronic illness show some additional reduction in the distribution of connectivity among functional links that have direct structural paths (*P* = 0.08, *d* = 0.44). Our findings address a critical gap in the literature linking structure and function in schizophrenia, and demonstrate for the first time that the abnormal state of functional connectivity preferentially affects structurally unconstrained links in schizophrenia. It also raises the question of a continuum of dysconnectivity ranging from less direct (structurally unconstrained) to more direct (structurally constrained) brain pathways underlying the progressive clinical staging and persistence of schizophrenia. *Hum Brain Mapp 36:4529–4538, 2015*. © **2015 The Authors. Human Brain Mapping Published by Wiley Periodicals, Inc.**

## INTRODUCTION

Optimal function of the human brain relies on the cooperation of constituent brain regions. The degree of cooperative activity between two brain regions at rest, measured using functional connectivity [Friston, 1994], varies greatly across the brain. A complete lack of spatial variability in functional connectivity indicates that the entire brain is either acting as a single homogenous unit without regional specialization, or the constituent brain regions are entirely asynchronous, without integration. In contrast, optimum spatial variability in connectional strength reflects simultaneous integration and segregation across distributed brain regions. This diversity forms the core of the overall topology of the complex functional architecture of human brain [Bullmore and Sporns, 2009; Tononi et al., 1994] altered in neuropsychiatric disorders such as schizophrenia [Alexander‐Bloch et al., 2010; Fornito et al., 2012; Rubinov and Bullmore, 2013; van den Heuvel et al., 2013].

In healthy controls, presence of structural connectivity (putative axonal linkage assessed using diffusion tensor imaging) strongly predicts the strength of functional connectivity [Damoiseaux and Greicius, 2009; Honey et al., 2009; Skudlarski et al., 2010; van den Heuvel et al., 2009]. Nevertheless, most of the pairwise functional connections exist in the absence of a direct axonal linkage between two regions [Adachi et al., 2011; Damoiseaux and Greicius, 2009; Honey et al., 2009]. In general, functional links that have a structural basis are stronger and involve anatomically more proximal regions [Honey et al., 2009]. In contrast, functional links between regions that do not share direct axonal linkage appear to be weaker, and involve spatially distant regions [Adachi et al., 2011; Honey et al., 2009]. Functional links in the absence of one‐to‐one axonal connections may emerge from directed polysynaptic connections or shared inputs/outputs involving a third region [Adachi et al., 2011]. Such indirect, weaker functional links may represent a connectional architecture that is physiologically distinct from the links with a more direct structural basis.

Several studies observe a reduction in overall strength of functional connectivity in schizophrenia [Argyelan et al., 2013; Bassett et al., 2012; Lynall et al., 2010], while both increased [Skudlarski et al., 2010; Whitfield‐Gabrieli et al., 2009] and decreased [Bluhm et al., 2007; Liang et al., 2006] connectivity involving different regional connections are noted across the brain [Karbasforoushan and Woodward, 2012; Pettersson‐Yeo et al., 2011; Rubinov and Bullmore, 2013]. The presence of both hyper‐ and hypoconnectivity involving different regional connections [Guo et al., 2013; Skudlarski et al., 2010; Venkataraman et al., 2012; Woodward et al., 2012] indicates a large diversity in the distribution of connectivity across the functional links in schizophrenia. If such a diversification lies at the core of the dynamic pathophysiological process defining the presence of schizophrenia in an individual, then for a randomly chosen pair of brain regions, the connectional strength is likely to be less predictable in a patient compared to a healthy control.

The unpredictability of bivariate functional connectivity across the entire brain can be quantified using an index of entropy based on the principles of information theory [Shannon, 1948]. Functional Connectivity Entropy (FCE) is a measure of randomness of the strength of functional connectivity, across all possible pairwise interregional connections in the brain [Yao et al., 2013]. This is different from the unpredictability or complexity across time that has been investigated previously in schizophrenia [Bassett et al., 2012; Fernández et al., 2013]. Higher FCE represents the presence of increased variability in connectional strength and has been observed in association with healthy ageing [Yao et al., 2013]. On the basis of a computational model, altered FCE has been attributed to a reduction in the pool of excitatory neurons [Yao et al., 2013], an observation that is highly relevant to the study of schizophrenia [Anticevic et al., 2013].

Recent observations also suggest that weak (rather than strong) [Bassett et al., 2012] and long‐distance (rather than short distance) functional links [Guo et al., 2013] are crucial to discriminate patients with schizophrenia from controls. This raises the possibility that the connectional architecture involving functional links that have no direct structural connectivity may be preferentially affected in schizophrenia.

Here, we tested the hypothesis that the randomness of functional connectivity (measured using FCE) is abnormal in schizophrenia, and this abnormality is specific to functional links with no direct structural connectivity. We also investigated the relationship between FCE and symptom burden in patients. Given its onset around adolescence, various dynamic changes coinciding with brain maturation take place in the initial few years in the course of schizophrenia, giving rise to several unstable and inconsistent neurobiological patterns that stabilized during the later stage of the illness [Pantelis et al., 2009]. In particular, the architecture of functional connectivity in patients is significantly affected by duration of illness. Patients with longer duration of illness show reduced segregation and integration [Liu et al., 2008], and reduced connectivity among core brain hubs [Collin et al., 2013]. In light of these observations, we expected a moderating effect of illness duration on FCE.

## METHODS

### Participants

This sample has been previously reported in our earlier study [Palaniyappan et al., 2013]. Thirty‐four patients satisfying DSM‐IV criteria for schizophrenia (*n* = 28) or schizoaffective disorder (*n* = 6) and 32 age, gender and parental socio‐economic status [Rose and Pevalin, 2003] matched healthy controls were included. Patients were recruited from the community‐based mental health teams (including Early Intervention in Psychosis teams) in Nottinghamshire and Leicestershire, UK. The diagnosis was made in a clinical consensus meeting in accordance with the procedure of Leckman et al. [1982], using all available information including a review of case files and a standardized clinical interview (SSPI) [Liddle et al., 2002]. All patients were in a stable phase of illness (defined as a change of no more than ten points in their Global Assessment of Function score, assessed 6 weeks prior and immediately prior to study participation). The study was given ethical approval by the National Research Ethics Committee, Derbyshire, UK. All volunteers gave written informed consent. Clinical and demographic characteristics of this sample are presented in Table 1.

**Table 1:** Clinical and demographic features

	Patients (*n* = 34)	Controls (*n* = 32)	*t*/*X* ^2^, *P*
Age	34.1 ± 9.1	33.4 ± 9.1	*t* = 0.30, *P* = 0.76
Gender	25/9	22/10	*χ*2 = 0.18, *P* = 0.67
Mean parental NS‐SEC (SD)	2.45 ± 1.5	2.22 ± 1.4	*t* = 0.24, *P* = 0.51
Handedness (right/left)	29/5	28/4	*χ*2 = 0.07, *P* = 0.79
SOFAS score	54.4 ± 13.2	–	–
Antipsychotic dose (CPZ equivalents)	694.5 ± 715.8	–	–
Median duration of illness in years (range)	6.0 (28)		
Total SSPI score	11.8 ± 7.7	–	–

Patients were interviewed on the same day as the scan by a research psychiatrist (LP and VB) and clinical severity of psychosis over the previous week was assessed on the basis of total SSPI scores. Duration of illness was estimated from the time of reported onset of psychotic symptoms (Criterion A of DSM‐IV schizophrenia), on the basis of information from case notes and clinical interview. We divided the patient sample into early stage (<5 years duration) and later‐stage (>5 years) illness, based on the clinical notion of “critical period” of psychosis during which interventions can modify outcome [Crumlish et al., 2009; McGorry, 2002; McGorry et al., 2008] and clinical stability is achieved [Bleuler, 1978]. Thirty‐two out of 34 patients were receiving antipsychotic treatment at the time of scan. The chlorpromazine equivalent doses were calculated using data presented by Woods [2003] and Chong et al. [2000] (for clozapine). In addition, 25 mg risperidone depot injection every 14 days was considered equivalent to 4 mg oral risperidone per day, in accordance with the recommendation of the British National Formulary [Joint Formulary Committee, 2013].

## MRI ACQUISITION

All scans were conducted at Sir Peter Mansfield Magnetic Resonance Centre, University of Nottingham using 3T Philips Achieva MR Scanner (Philips Medical Systems, The Netherlands). A structural T1 image was acquired along with a series of diffusion‐weighted images and 10‐min long eyes‐closed resting‐state functional MRI in the same scanning session. Details of the acquisition are presented in the Supporting Information Material SM1.

## DTI PROCESSING

For DTI images, we first used FMRIB Software Library v5.0 (http://fsl.fmrib.ox.ac.uk/fsl) [Jenkinson et al., 2012] to remove the eddy‐current and extract the brain mask from the B0 image. Then, we used TrackVis [Wang et al., 2007] to obtain the fiber images by the deterministic tracking method, with anatomical regions defined using the automated anatomical labelling atlas (AAL) convention on the basis of coregistered T1 image from each subject. This enabled us to determine the presence of streamlines connecting every pair of brain regions. All the processes were performed using the PANDA suite [Cui et al., 2013].

If two brain regions A and B have one or more streamlines directly connecting each other, then this link AB is regarded as a direct or primary structural path. In contrast, if two brain regions X and Y have no direct connections, but share a direct connection with a common third region Z, then the link XY can be regarded as indirect secondary structural path. If two brain regions K and L have neither direct connections, nor a common third region, but are connected to each other indirectly by virtue of being connected a common direct link (for example, K connects directly to A, A connects directly to B, B connects directly to L), then the link KL can be regarded as indirect tertiary structural path. To study the effect of the minimum streamline threshold, we varied the minimum from one to three and repeated the primary analysis (group comparisons). The results, shown in Supporting Information Material 6, indicated that the structural paths were robust across these thresholds.

**Figure 1:**
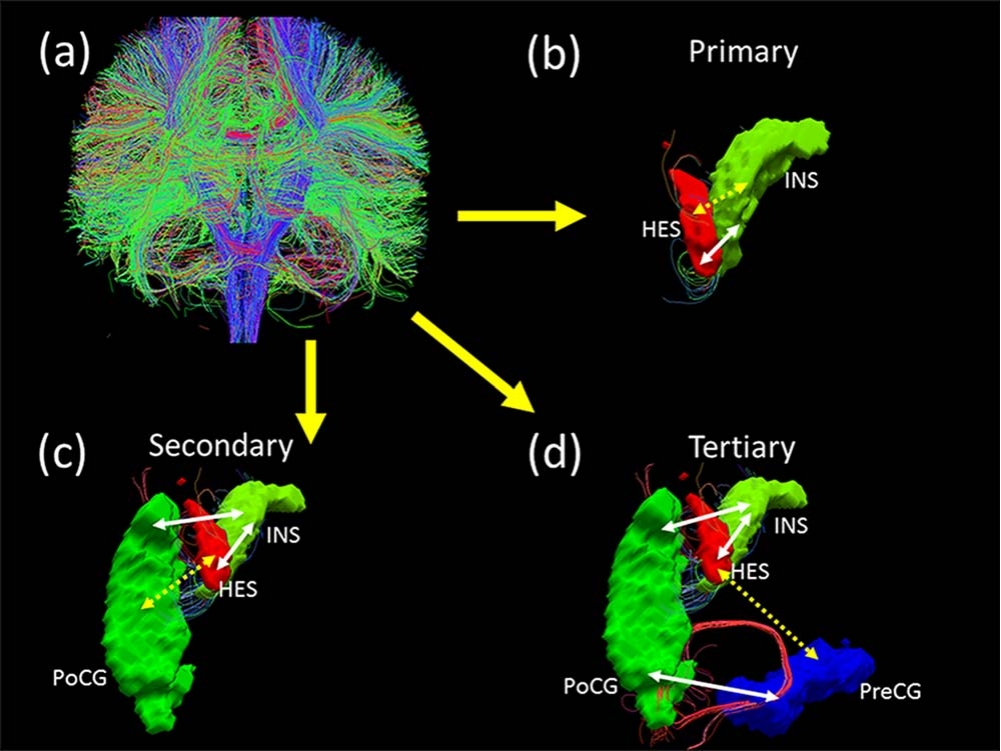
**Three types of structural paths defined using diffusion tractography.** (**a**) whole‐brain fiber connection network construction by Diffusion Tensor Imaging data; (**b**) Heschl gyrus (HES) and Insula (INS) are directly connected with each other through white matter tracts. HES↔INS link is a primary structural path. (**c**) Heschl gyrus and Postcentral gyrus (PoCG) are indirectly linked (discontinuous arrow) through white matter tracts (continuous arrows) that connect HES and PoCG with the Insula. Though there are no direct connections between HES and PoCG, INS acts as a common third region. HES ↔ PoCG link is termed as a secondary structural path. (**d**) Heschl gyrus and Precentral gyrus (PreCG) are indirectly linked (discontinuous arrow) through white matter tracts (continuous arrows) that connect HES with INS, INS with PoCG and PoCG with PreCG. There are no direct connections or a single common third region between HES and PreCG. HES ↔ PreCG link is termed as a tertiary structural path. [Color figure can be viewed in the online issue, which is available at http://wileyonlinelibrary.com.]

Thus, for every subject, we identified primary, secondary, and tertiary paths on the basis of the DTI fiber streamlines. An illustration of these paths is shown in Figure 1.

### fMRI Processing

Weighted summation of the dual‐echo images produced a single set of low‐artefact functional images [Posse et al., 1999]. Retrospective physiological correction was then performed [Glover et al., 2000]. The first five volumes of fMRI datasets were discarded, to allow for scanner stabilization. Further processing was then conducted by Statistical Parametric Mapping (SPM8) (http://www.fil.ion.ucl.ac.uk/spm) [Penny et al., 2011] and Data Processing Assistant for Resting‐State fMRI [Chao‐Gan and Yu‐Feng, 2010]. After slice‐timing correction and realignment to the middle volume, the functional scans were spatially normalized using the unified segmentation approach to a standard template (Montreal Neurological Institute) and resampled to 3 × 3 × 3 mm^3^. Data was then smoothed using a Gaussian kernel of 8 mm full‐width at half‐ maximum, detrended, and then passed through a band‐pass filter (0.01–0.08 Hz) to reduce low‐frequency drift and high‐frequency physiological noise.

Nuisance covariates including head motion, global mean signals, white matter signals and cerebrospinal signals were regressed out from the timeseries. Regional time series were extracted in each of the 90 AAL [Tzourio‐Mazoyer et al., 2002] based brain regions by averaging the signals of all voxels within a region. The names of the 90 brain regions are listed in Supporting Information Table ST1, along with further details of fMRI preprocessing in SM2, SF1 and the characteristics of excluded subjects in SM3.

## STATISTICAL INFERENCE

We applied linear regression [Seber and Lee, 2012] to remove age, sex and dose effects for patients and controls. We used Cohen's d [Cohen, 1988] to quantify the effect size of the mean differences.

## RESULTS

### Structural Paths

Using the path definitions based on DTI, we noted that in 96% of subjects (64 out of 66), all 4,005 possible structural links could be classified into primary, secondary or tertiary structural paths. In 27 subjects (14 patients and 13 controls), there were no more than seven pairs of regions that required more than two intermediate linked structures to form an indirect path. On average we obtained 27.8% primary, 68.9% secondary, and 3.4% tertiary structural paths in controls. In patients, 27.4% primary, 69.5% secondary and 3.1% tertiary structural paths were noted, with no significant difference between the two groups (Supporting Information Figure SF2). Of the 4005 possible connections, 3774 were in the same category (primary, secondary, or tertiary) when comparing patients and controls. A substantial degree of agreement was noted across the three categories of connections when the two groups of subjects were compared. 95.4% of primary, 91.5% of secondary, and 75.6% links are classified in the same group in both patients and controls.

We also examined the anatomical distribution of the primary, secondary, and tertiary paths using the conventional distribution of Resting State Networks observed in previous studies. This is presented in Supporting Information 2.

### Changes in FCE

Patients did not significantly differ in the whole brain FCE from controls when considered as a single group (*P* = 0.5). But when stratified according to illness duration, whole brain FCE was significantly higher in patients with early stage illness (*P* = 0.01, *d* = 0.91) and lower in those with chronic illness (*P* = 2 × 10^−6^, *d* = 1.69) in comparison with age and sex‐matched controls.

**Figure 2:**
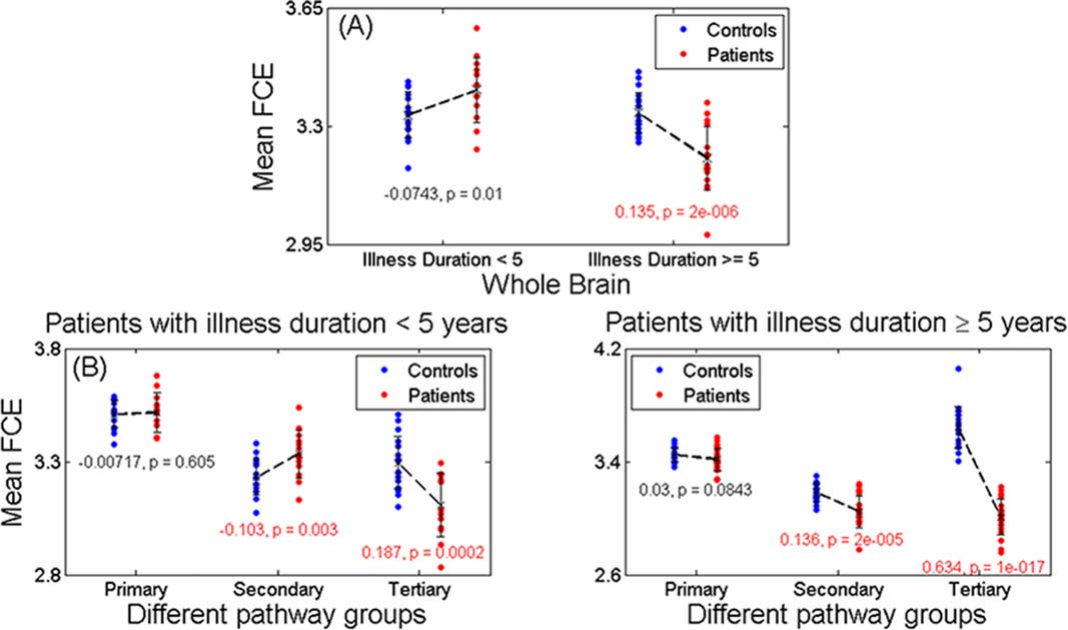
FCE in different fiber pathway. (**a**): In patients < 5 years, the FCE was slightly increased in patients (0.0743, *P* = 0.01, *d* = 0.91), and significantly decreased (0.135, *P* = 2 × 10^−16^, *d* = 1.69) in patients ≥ 5 years comparing with controls. (**b**): Left panel: In patients < 5 years, the functional connectivity (FCE) entropy was almost unchanged (−0.00717, *P* = 0.605, *d* = 0.10) in primary pathways, significantly increased (−0.103, *P* = 0.003, *d* = 1.12) in secondary ones and significantly decreased (0.187, *P* = 0.0002, *d* = 1.46) in tertiary ones comparing with controls. Right panel: In patients ≥ 5 years, the FCE was close to significantly decreased (0.03, *P* = 0.0843, *d* = 0.44) in primary pathways, significantly decreased (0.136, *P* = 2 × 10^−5^, *d* = 1.47) in secondary and most significantly decreased (0.634, *P* = 1 × 10^−17^, *d* = 4.66) in tertiary when compared with controls. All the effects of age, sex, and dose have been removed. [Color figure can be viewed in the online issue, which is available at http://wileyonlinelibrary.com.]

Both patients in the early stage of schizophrenia (*P* = 0.0002, *d* = 1.46) and chronic illness (*P* = 1 × 10^−17^, *d* = 4.66) showed a showed a significant reduction in the FCE of tertiary paths when compared to age and sex‐matched controls. FCE of secondary paths was increased in those with early stage schizophrenia (*P* = 0.003, *d* = 1.12), but significantly reduced in patients at a more chronic stage of illness (*P* = 2 × 10^−5^, *d* = 1.47). FCE of primary paths showed a trend towards an increase in patients with chronic illness (*P* = 0.084, *d* = 0.44) but not in those with early stage illness when compared to matched controls (Fig. 2 panel B).

**Figure 3:**
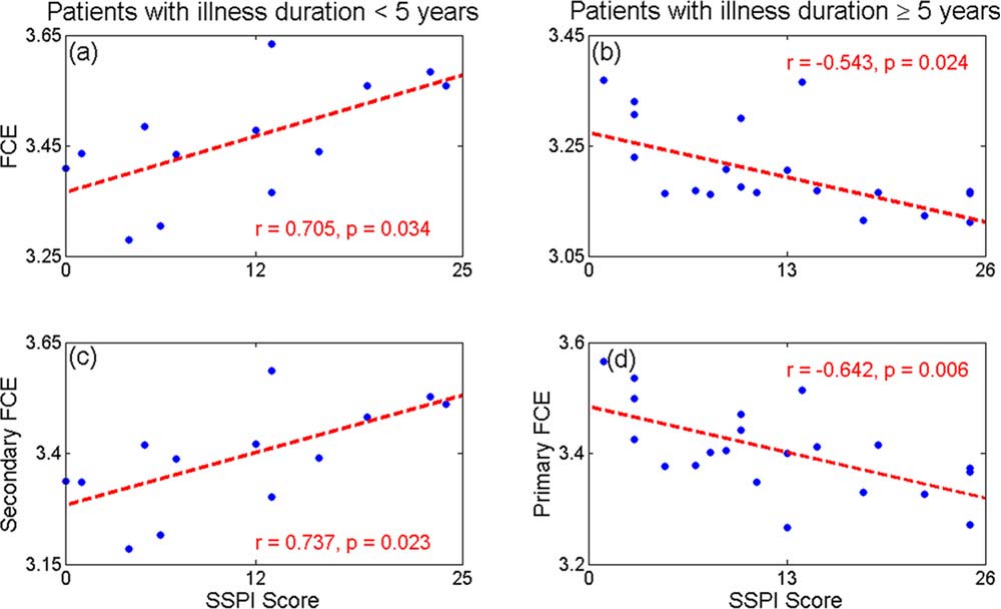
Functional entropy VS SSPI score. Panel (**a**, **b**): The FCE held a significantly (*r* = 0.705, *P* = 0.034) positive correlation with SSPI score in patients < 5 years (left panel), while it displayed a significantly (*r* = −0.543, *P* = 0.024) negative correlation in those ≥ 5 years (right panel). Panel (**c**, **d**): The secondary FCE showed a significant (*r* = 0.737, *P* = 0.023) positive correlation with SSPI score in patients < 5 years (left panel), while the primary functional entropy displayed a significant (*r* = −0.642, *P* = 0.006) negative correlation in those ≥ 5 years (right panel). Linear effects of age, sex, and dose have been removed. [Color figure can be viewed in the online issue, which is available at http://wileyonlinelibrary.com.]

In the early stage of schizophrenia, higher whole brain FCE was associated with higher symptom burden measured using the total SSPI score (*r* = 0.705, *P* = 0.034); this was mostly driven by the increased FCE of secondary paths which was significantly related to the symptom burden(*r* = 0.74, *P* = 0.023), while the FCE of primary and tertiary paths did not show any significant relationships. In those with chronic illness, lower whole brain FCE was associated with higher symptom burden (*r* = −0.543, *P* = 0.024); this was driven by the FCE of both primary (*r* = −0.64, *P* = 0.006) and secondary paths (*r* = −0.51, *P* = 0.036) being negatively correlated with the symptom burden, while tertiary paths showed no significant relationship (Fig. 3).

## DISCUSSION

When compared with healthy controls, the whole brain FCE is higher in the early stages of schizophrenia indicating an increase in unpredictability and spatial randomness of functional connectivity, especially in those with more severe illness. A pronounced reduction in whole‐brain FCE is seen in the later stages of schizophrenia, indicating that the underlying pathophysiological process may be associated with an indistinct, nonspecific mode of brain operation at rest. As hypothesized originally, we note that the most significant changes in the randomness of functional connectivity in schizophrenia occur in indirect paths that do not have a direct axonal linkage. In particular, we note that the variability of connectivity in tertiary paths shows a striking reduction in both early and late stages of schizophrenia, not altered by the severity of symptoms, suggesting that this is an invariant, “trait‐like” feature of the illness.

FCE is reduced in tertiary paths in both groups, and across all pathways and in the whole brain in those with chronic illness. One exception to the observation of predominant FCE reduction is the increased FCE in secondary paths that annuls the effect of FCE reduction seen in tertiary links, contributing to a lack of group difference with controls, when whole brain FCE is considered in the early stage illness. One may speculate that the increase in FCE seen in the secondary paths in early stages may reflect a compensatory effort against the process driving a predominant reduction in FCE; the observation that those with more severe illness in early stages show a higher FCE in secondary paths, supports this speculation.

Patients with chronic schizophrenia showed reduced variability in secondary paths, with a trend towards reduced variability in primary paths as well. Both of these changes in the primary (most significant) and secondary paths were more pronounced in those with more severe illness. One may speculate, on this basis, that any compensatory increase in FCE seen in secondary paths at early stages, “decays” over the course of the illness as the process of FCE reduction now extends to the secondary and primary pathways in those with a more chronic illness. On the basis of this observation, we speculate that in the backdrop of pervasive alteration in tertiary (highly indirect) links, the dynamic pathophysiological process that contributes to clinical severity may progressively “penetrate” the less indirect (secondary) and more direct (primary) pathways. As this sample is not longitudinal, we are not able to draw any firm conclusions on the progressive nature of these changes. Nevertheless, we can infer a continuum of dysconnectivity on the axis of anatomical connectedness associated with varied clinical severity and persistence of schizophrenia.

FCE uniquely captures the uncertainty introduced by bidirectional deviations (both increase and decrease) of connectivity strength occurring in schizophrenia. It provides an intuitive measure of the overall randomness in the distribution of connectivity, irrespective of the mean strength of the functional links, changes in which can be nullified by the bidirectional changes across the brain. Though FCE has not been previously employed in the investigation of schizophrenia, the issue of spatial diversity of functional connections was investigated in two previous studies. Both Lynall et al., [2010] and Bassett et al., [2012] quantified the average of the variance in the pairwise connections within each brain region (column‐wise variance across brain regions in the functional connectivity matrix) and reported an increase in the dispersion of functional connectivity in schizophrenia. Unlike variance, entropy is an index of diversification and uncertainty that does not depend on the absolute values of the underlying metric (in this case, functional connectivity), and uses more information about the probability distribution than variance, especially relevant in the presence of fat‐tailed distributions [Schwarz and McGonigle, 2011; Skudlarski et al., 2010]. Higher entropy within a system suggests that the whole provides more information than the sum of its parts [Tononi et al., 1994]. Variance does not capture the unpredictability when the values taken by a variable are similar to each other (i.e., narrow distribution range) [Bach and Dolan, 2012]. FCE is a more intuitive index of uncertainty when a subtle randomization is expected, as in schizophrenia. Our observations suggest that FCE is a relevant measure in this context that is sensitive to both the clinical stage and severity of schizophrenia.

Unlike the indirect paths, the primary structural paths showed no significant alteration in the early stages and only a mild reduction in FCE reaching trend level significance in the chronic illness. If one assumes that the pathophysiology of schizophrenia primarily affects the functional relationship among brain regions, then this observation suggests that the relationship among brain regions that have a direct axonal linkage is less susceptible, at least in the early stages of the illness. In fact, several crucial alterations in functional connectivity relevant to the pathophysiology of schizophrenia have been noted between regions that do not have direct anatomical linkage. To consider an example, consistent functional connectivity between insula and anterior cingulate cortex has been observed using resting state fMRI, constituting a Salience Network [Menon and Uddin, 2010; Seeley et al., 2007; Taylor et al., 2009]. But to date, no consistent direct axonal linkage has been noted between these two regions [Cerliani et al., 2012; Cloutman et al., 2012; van den Heuvel et al., 2009]. Several studies have now shown that the abnormalities within this “indirect” functional network, is a key feature of psychosis [Manoliu et al., 2013; Palaniyappan et al., 2013; Pu et al., 2012; White et al., 2010]. As shown in the Supporting Information 1, the largest proportion of tertiary paths involved a network that included the insula. Focused investigation of the anatomical distribution of indirect functional links is warranted in future studies.

Poor spatial coherence between structural and functional connectivity has been previously reported in schizophrenia [Skudlarski et al., 2010], implying functional dysconnectivity involving anatomically unconnected regions. Indirect paths indicate the presence of weaker links in the functional connectivity architecture [Goni et al., 2014]. While most previous investigations of the connectome used thresholding procedures to discarded weaker links from analysis, the importance of weak links is being increasingly appreciated in recent times [Gallos et al., 2012; Schwarz and McGonigle, 2011]. The integrity of weaker links relate to global cognitive capacity in healthy individuals [Santarnecchi et al., 2014]. Bassett et al. [2012] have also shown that in schizophrenia abnormalities involving the weaker links are likely to be more specific to the illness process and relate to both cognitive abnormalities and symptom severity in schizophrenia. Our results also suggest that while there is a substantial overlap in the anatomical similarity of primary and secondary paths between patients and controls, 24.4% of all identified tertiary paths were present only in one group of subjects, highlighting that the weaker, indirect links are more prone to spatial redistribution, in addition to reduced variability in the strength of connectivity.

Functional connectivity of secondary but not tertiary paths can be attributed to connections mediated by a third region either by directed polysynaptic linkage [Lu et al., 2011] or shared afferents [Adachi et al., 2011]. As tertiary pathways do not share common afferent/efferent links, the observed connectivity in these paths are likely to be related to the emergent properties of the network‐level architecture [Adachi et al., 2011; Goni et al., 2014]. Uncovering the processes beyond physical connections that bring about functional correlation across time to “bind” a distributed set of brain regions is likely to be a crucial step to understand the pathophysiology of psychosis.

The novelty of our study includes the categorization of functional links using DTI‐based structural paths, the measurement of an unbiased estimate of unpredictability associated with functional connectivity, and the investigation of the effect of illness stage and severity on the structure‐function relationship. Several limitations must be considered when interpreting our observations. To study the effect of illness duration, we used an arbitrary cut‐off point of 5 years. From the multitude of neuroimaging studies investigating the effect of illness duration, no single period has emerged as the time of stabilization of brain changes after the onset. Nevertheless, existing evidence suggests that amount of dynamic (supposedly progressive) changes are most pronounced immediately before onset and in the first five years of illness. We studied a medicated sample of patients; antipsychotic treatment alters functional connectivity of brain regions in schizophrenia [Bolding et al., 2012; Stephan et al., 2001]. Given the suspected confound, we adjusted for the current dose of prescribed antipsychotics. We did not have information on cumulative antipsychotic exposure, but unlike volumetric changes that become more pronounced with cumulative exposure, functional dysconnectivity normalizes or returns to baseline levels on using antipsychotics [Abbott et al., 2013]. As a result, functional dysconnectivity in medicated samples are likely to be an underestimate of the true effect. In the absence of investigations focusing on the effect of antipsychotics on indirect functional links, caution is warranted when interpreting these results. Further, when computing the spatial variability of functional connectivity, we have assumed stationarity of the functional architecture within the time scale of fMRI. The unpredictability introduced by nonstationarity (dynamic “making and breaking” of functional dependencies) [Messe et al., 2014] is beyond the scope of this work.

## CONCLUSION

To conclude, in a healthy brain, the resting connectional architecture (system as a whole) accommodates a higher degree of variability in functional connections (constituent parts), but in established schizophrenia the variability of constituents that make up this system is limited. Our findings address a critical gap in the literature linking structure and function in schizophrenia, and demonstrate for the first time that the abnormal state of functional connectivity preferentially affects structurally unconstrained links in schizophrenia. It also raises the question of a continuum of dysconnectivity ranging from less direct (structurally unconstrained) to more direct (structurally constrained) brain pathways underlying the progressive clinical stages of schizophrenia.

## Supporting information

Supporting Information

Supporting Information

## References

[hbm22932-bib-0001] Abbott C , Jaramillo A , Wilcox C , Hamilton D (2013): Antipsychotic drug effects in schizophrenia: a review of longitudinal FMRI investigations and neural interpretations. Curr Med Chem 20:428 23157635 10.2174/0929867311320030014 PMC3632416

[hbm22932-bib-0002] Adachi Y , Osada T , Sporns O , Watanabe T , Matsui T , Miyamoto K , Miyashita Y. (2011): Functional connectivity between anatomically unconnected areas is shaped by collective network‐level effects in the macaque cortex. Cerebral Cortex 22:1586–1592. 21893683 10.1093/cercor/bhr234

[hbm22932-bib-0003] Alexander‐Bloch AF , Gogtay N , Meunier D , Birn R , Clasen L , Lalonde F , Lenroot R , Giedd J , Bullmore ET (2010): Disrupted modularity and local connectivity of brain functional networks in childhood‐onset schizophrenia. Front Syst Neurosci 4. 10.3389/fnsys.2010.00147 PMC2965020 21031030

[hbm22932-bib-0004] Anticevic A , Cole MW , Repovs G , Savic A , Driesen NR , Yang G , Cho YT , Murray JD , Glahn DC , Wang X‐J (2013): Connectivity, pharmacology, and computation: Toward a mechanistic understanding of neural system dysfunction in schizophrenia. Front Psychiatry 4:1–21. 24399974 10.3389/fpsyt.2013.00169 PMC3871997

[hbm22932-bib-0005] Argyelan M , Ikuta T , DeRosse P , Braga RJ , Burdick KE , John M , Kingsley PB , Malhotra AK , Szeszko PR (2013): Resting‐state FMRI connectivity impairment in schizophrenia and bipolar disorder. Schizoph Bull 40:100–110. 23851068 10.1093/schbul/sbt092 PMC3885301

[hbm22932-bib-0006] Bach DR , Dolan RJ (2012): Knowing how much you don't know: A neural organization of uncertainty estimates. Nat Rev Neurosci 13:572–586. 22781958 10.1038/nrn3289

[hbm22932-bib-0007] Bassett DS , Nelson BG , Mueller BA , Camchong J , Lim KO (2012): Altered resting state complexity in schizophrenia. Neuroimage 59:2196–2207. 22008374 10.1016/j.neuroimage.2011.10.002 PMC3254701

[hbm22932-bib-0008] Bleuler M. 1978 The schizophrenic disorders: Long‐term patient and family studies. Yale University Press, New Haven, USA.

[hbm22932-bib-0009] Bluhm RL , Miller J , Lanius RA , Osuch EA , Boksman K , Neufeld R , Théberge J , Schaefer B , Williamson P (2007): Spontaneous low‐frequency fluctuations in the BOLD signal in schizophrenic patients: Anomalies in the default network. Schizoph Bull 33:1004–1012. 17556752 10.1093/schbul/sbm052 PMC2632312

[hbm22932-bib-0010] Bolding MS , White DM , Hadley JA , Weiler M , Holcomb HH , Lahti AC (2012): Antipsychotic drugs alter functional connectivity between the medial frontal cortex, hippocampus, and nucleus accumbens as Measured by H215O PET. Front Psychiatry 3:105 23230425 10.3389/fpsyt.2012.00105 PMC3515723

[hbm22932-bib-0011] Bullmore E , Sporns O (2009): Complex brain networks: Graph theoretical analysis of structural and functional systems. Nat Rev Neurosci 10:186–198. 19190637 10.1038/nrn2575

[hbm22932-bib-0012] Cerliani L , Thomas RM , Jbabdi S , Siero JC , Nanetti L , Crippa A , Gazzola V , D'Arceuil H , Keysers C (2012): Probabilistic tractography recovers a rostrocaudal trajectory of connectivity variability in the human insular cortex. Hum Brain Mapp 33:2005–2034. 21761507 10.1002/hbm.21338 PMC3443376

[hbm22932-bib-0013] Chao‐Gan Y , Yu‐Feng Z (2010): DPARSF: A MATLAB toolbox for “pipeline” data analysis of resting‐state fMRI. Front Syst Neurosci 4. 10.3389/fnsys.2010.00013 PMC2889691 20577591

[hbm22932-bib-0014] Chong S‐A , Remington GJ , Bezchlibnyk‐Butler KZ (2000): Effect of clozapine on polypharmacy. Psychiatr Serv 51:250–252. 10655014 10.1176/appi.ps.51.2.250

[hbm22932-bib-0015] Cloutman LL , Binney RJ , Drakesmith M , Parker GJ , Lambon Ralph MA (2012): The variation of function across the human insula mirrors its patterns of structural connectivity: Evidence from in vivo probabilistic tractography. Neuroimage 59:3514–3521. 22100771 10.1016/j.neuroimage.2011.11.016

[hbm22932-bib-0016] Cohen J (1988): Statistical power analysis for the behavioral sciences. Psychology Press, London, UK.

[hbm22932-bib-0017] Collin G , Kahn RS , de Reus MA , Cahn W , van den Heuvel MP (2013): Impaired rich club connectivity in unaffected siblings of schizophrenia patients. Schizophr Bull 40:438–448. 24298172 10.1093/schbul/sbt162 PMC3932089

[hbm22932-bib-0018] Crumlish N , Whitty P , Clarke M , Browne S , Kamali M , Gervin M , McTigue O , Kinsella A , Waddington JL , Larkin C (2009): Beyond the critical period: Longitudinal study of 8‐year outcome in first‐episode non‐affective psychosis. Br J Psychiatry 194:18–24. 19118320 10.1192/bjp.bp.107.048942

[hbm22932-bib-0019] Cui Z , Zhong S , Xu P , He Y , Gong G (2013): PANDA: A pipeline toolbox for analyzing brain diffusion images. Front Human Neurosci 7. 10.3389/fnhum.2013.00042 PMC3578208 23439846

[hbm22932-bib-0020] Damoiseaux JS , Greicius MD (2009): Greater than the sum of its parts: A review of studies combining structural connectivity and resting‐state functional connectivity. Brain Struct Funct 213:525–533. 19565262 10.1007/s00429-009-0208-6

[hbm22932-bib-0021] Fernández A , Gómez C , Hornero R , López‐Ibor JJ (2013): Complexity and schizophrenia. Prog Neuropsychopharmacol Biol Psychiatry 45:267–276. 22507763 10.1016/j.pnpbp.2012.03.015

[hbm22932-bib-0022] Fornito A , Zalesky A , Pantelis C , Bullmore ET (2012): Schizophrenia, neuroimaging and connectomics. Neuroimage 62:2296–2314. 22387165 10.1016/j.neuroimage.2011.12.090

[hbm22932-bib-0023] Friston KJ (1994): Functional and effective connectivity in neuroimaging: A synthesis. Human Brain Mapp 2:56–78.

[hbm22932-bib-0024] Gallos LK , Makse HA , Sigman M (2012): A small world of weak ties provides optimal global integration of self‐similar modules in functional brain networks. Proc Natl Acad Sci 109:2825–2830. 22308319 10.1073/pnas.1106612109 PMC3286928

[hbm22932-bib-0025] Glover GH , Li TQ , Ress D (2000): Image‐based method for retrospective correction of physiological motion effects in fMRI: RETROICOR. Magn Reson Med 44:162–167. 10893535 10.1002/1522-2594(200007)44:1<162::aid-mrm23>3.0.co;2-e

[hbm22932-bib-0026] Goni J , van den Heuvel MP , Avena‐Koenigsberger A , Velez de Mendizabal N , Betzel RF , Griffa A , Hagmann P , Corominas‐Murtra B , Thiran JP , Sporns O (2014): Resting‐brain functional connectivity predicted by analytic measures of network communication. Proc Natl Acad Sci U S A 111:833–838. 24379387 10.1073/pnas.1315529111 PMC3896172

[hbm22932-bib-0027] Guo S , Palaniyappan L , Yang B , Liu Z , Xue Z , Feng J (2013): Anatomical distance affects functional connectivity in patients with schizophrenia and their siblings. Schizophr Bull 40:449–459. 24282323 10.1093/schbul/sbt163 PMC3932090

[hbm22932-bib-0028] Honey C , Sporns O , Cammoun L , Gigandet X , Thiran J‐P , Meuli R , Hagmann P (2009): Predicting human resting‐state functional connectivity from structural connectivity. Proc Natl Acad Sci 106:2035–2040. 19188601 10.1073/pnas.0811168106 PMC2634800

[hbm22932-bib-0029] Jenkinson M , Beckmann CF , Behrens TE , Woolrich MW , Smith SM (2012): Fsl. Neuroimage 62:782–790. 21979382 10.1016/j.neuroimage.2011.09.015

[hbm22932-bib-0030] Joint Formulary Committee (2013): British national formulary. Pharmaceutical Press, London, UK.

[hbm22932-bib-0031] Karbasforoushan H , Woodward N (2012): Resting‐state networks in schizophrenia. Curr Top Med Chem 12:2404–2414. 23279179 10.2174/156802612805289863

[hbm22932-bib-0032] Leckman JF , Sholomskas D , Thompson D , Belanger A , Weissman MM (1982): Best estimate of lifetime psychiatric diagnosis: A methodological study. Arch Gen Psychiatry 39:879–883. 7103676 10.1001/archpsyc.1982.04290080001001

[hbm22932-bib-0033] Liang M , Zhou Y , Jiang T , Liu Z , Tian L , Liu H , Hao Y (2006): Widespread functional disconnectivity in schizophrenia with resting‐state functional magnetic resonance imaging. Neuroreport 17:209–213. 16407773 10.1097/01.wnr.0000198434.06518.b8

[hbm22932-bib-0034] Liddle PF , Ngan ET , Duffield G , Kho K , Warren AJ (2002): Signs and symptoms of psychotic illness (SSPI): A rating scale. Br J Psychiatry 180:45–50. 11772851 10.1192/bjp.180.1.45

[hbm22932-bib-0035] Liu Y , Liang M , Zhou Y , He Y , Hao Y , Song M , Yu C , Liu H , Liu Z , Jiang T (2008): Disrupted small‐world networks in schizophrenia. Brain 131:945–961. 18299296 10.1093/brain/awn018

[hbm22932-bib-0036] Lu J , Liu H , Zhang M , Wang D , Cao Y , Ma Q , Rong D , Wang X , Buckner RL , Li K (2011): Focal pontine lesions provide evidence that intrinsic functional connectivity reflects polysynaptic anatomical pathways. J Neurosci 31:15065–15071. 22016540 10.1523/JNEUROSCI.2364-11.2011 PMC3397237

[hbm22932-bib-0037] Lynall M‐E , Bassett DS , Kerwin R , McKenna PJ , Kitzbichler M , Muller U , Bullmore E (2010): Functional connectivity and brain networks in schizophrenia. J Neurosci 30:9477–9487. 20631176 10.1523/JNEUROSCI.0333-10.2010 PMC2914251

[hbm22932-bib-0038] Manoliu A , Riedl V , Doll A , Bäuml JG , Mühlau M , Schwerthöffer D , Scherr M , Zimmer C , Förstl H , Bäuml J (2013): Insular dysfunction reflects altered between‐network connectivity and severity of negative symptoms in schizophrenia during psychotic remission. Front Human Neurosci 7. 10.3389/fnhum.2013.00216 PMC3657709 23730284

[hbm22932-bib-0039] McGorry PD (2002): The recognition and optimal management of early psychosis: An evidence‐based reform. World Psychiatry 1:76–83. 16946857 PMC1489880

[hbm22932-bib-0040] McGorry PD , Killackey E , Yung A (2008): Early intervention in psychosis: Concepts, evidence and future directions. World Psychiatry 7:148–156. 18836582 10.1002/j.2051-5545.2008.tb00182.x PMC2559918

[hbm22932-bib-0041] Menon V , Uddin LQ (2010): Saliency, switching, attention and control: A network model of insula function. Brain Struct Funct 214:655–667. 20512370 10.1007/s00429-010-0262-0 PMC2899886

[hbm22932-bib-0042] Messe A , Rudrauf D , Benali H , Marrelec G (2014): Relating structure and function in the human brain: relative contributions of anatomy, stationary dynamics, and non‐stationarities. PLoS Comput Biol 10:e1003530 24651524 10.1371/journal.pcbi.1003530 PMC3961181

[hbm22932-bib-0043] Palaniyappan L , Simmonite M , White TP , Liddle EB , Liddle PF (2013): Neural primacy of the salience processing system in schizophrenia. Neuron 79:814–828. 23972602 10.1016/j.neuron.2013.06.027 PMC3752973

[hbm22932-bib-0044] Pantelis C , Yücel M , Bora E , Fornito A , Testa R , Brewer WJ , Velakoulis D , Wood SJ (2009): Neurobiological markers of illness onset in psychosis and schizophrenia: The search for a moving target. Neuropsychol Rev 19:385–398. 19728098 10.1007/s11065-009-9114-1

[hbm22932-bib-0045] Penny WD , Friston KJ , Ashburner JT , Kiebel SJ , Nichols TE (2011): Statistical Parametric Mapping: The Analysis of Functional Brain Images: The Analysis of Functional Brain Images. Academic Press, Waltham, USA.

[hbm22932-bib-0046] Pettersson‐Yeo W , Allen P , Benetti S , McGuire P , Mechelli A (2011): Dysconnectivity in schizophrenia: Where are we now? Neurosci Biobehav Rev 35:1110–1124. 21115039 10.1016/j.neubiorev.2010.11.004

[hbm22932-bib-0047] Posse S , Wiese S , Gembris D , Mathiak K , Kessler C , Grosse‐Ruyken M‐L , Elghahwagi B , Richards T , Dager SR , Kiselev VG (1999): Enhancement of BOLD‐contrast sensitivity by single‐shot multi‐echo functional MR imaging. Magn Resonance Med 42:87–97. 10.1002/(sici)1522-2594(199907)42:1<87::aid-mrm13>3.0.co;2-o 10398954

[hbm22932-bib-0048] Pu W , Li L , Zhang H , Ouyang X , Liu H , Zhao J , Li L , Xue Z , Xu K , Tang H (2012): Morphological and functional abnormalities of salience network in the early‐stage of paranoid schizophrenia. Schizophr Res 141:15–21. 22910405 10.1016/j.schres.2012.07.017

[hbm22932-bib-0049] Rose D , Pevalin DJ. 2003 A researcher's guide to the national statistics socio‐economic classification. SAGE Publications Ltd New York, USA. 10.1016/s0277-9536(00)00136-2 11005397

[hbm22932-bib-0050] Rubinov M , Bullmore E (2013): Fledgling pathoconnectomics of psychiatric disorders. Trends Cogn Sci 17:641–647. 24238779 10.1016/j.tics.2013.10.007

[hbm22932-bib-0051] Santarnecchi E , Galli G , Polizzotto NR , Rossi A , Rossi S (2014): Efficiency of weak brain connections support general cognitive functioning. Hum Brain Mapp 35:4566–4582. 24585433 10.1002/hbm.22495 PMC6869093

[hbm22932-bib-0052] Schwarz AJ , McGonigle J (2011): Negative edges and soft thresholding in complex network analysis of resting state functional connectivity data. Neuroimage 55:1132–1146. 21194570 10.1016/j.neuroimage.2010.12.047

[hbm22932-bib-0053] Seber GA , Lee AJ. 2012 Linear regression analysis. Wiley, New Jersey, USA.

[hbm22932-bib-0054] Seeley WW , Menon V , Schatzberg AF , Keller J , Glover GH , Kenna H , Reiss AL , Greicius MD (2007): Dissociable intrinsic connectivity networks for salience processing and executive control. J Neurosci 27:2349–2356. 17329432 10.1523/JNEUROSCI.5587-06.2007 PMC2680293

[hbm22932-bib-0055] Shannon C (1948): A mathematical theory of communication. Bell Syst Technol J 27:379

[hbm22932-bib-0056] Skudlarski P , Jagannathan K , Anderson K , Stevens MC , Calhoun VD , Skudlarska BA , Pearlson G (2010): Brain connectivity is not only lower but different in schizophrenia: A combined anatomical and functional approach. Biol Psychiatry 68:61–69. 20497901 10.1016/j.biopsych.2010.03.035 PMC2900394

[hbm22932-bib-0057] Stephan K , Magnotta V , White T , Arndt S , Flaum M , O'LEARY D , Andreasen N (2001): Effects of olanzapine on cerebellar functional connectivity in schizophrenia measured by fMRI during a simple motor task. Psychol Med 31:1065–1078. 11513374 10.1017/s0033291701004330

[hbm22932-bib-0058] Taylor KS , Seminowicz DA , Davis KD (2009): Two systems of resting state connectivity between the insula and cingulate cortex. Human Brain Mapp 30:2731–2745. 10.1002/hbm.20705 PMC6871122 19072897

[hbm22932-bib-0059] Tononi G , Sporns O , Edelman GM (1994): A measure for brain complexity: Relating functional segregation and integration in the nervous system. Proc Natl Acad Sci 91:5033–5037. 8197179 10.1073/pnas.91.11.5033 PMC43925

[hbm22932-bib-0060] Tzourio‐Mazoyer N , Landeau B , Papathanassiou D , Crivello F , Etard O , Delcroix N , Mazoyer B , Joliot M (2002): Automated anatomical labeling of activations in SPM using a macroscopic anatomical parcellation of the MNI MRI single‐subject brain. Neuroimage 15:273–289. 11771995 10.1006/nimg.2001.0978

[hbm22932-bib-0061] van den Heuvel MP , Mandl RC , Kahn RS , Pol H , Hilleke E (2009): Functionally linked resting‐state networks reflect the underlying structural connectivity architecture of the human brain. Human Brain Mapp 30:3127–3141. 10.1002/hbm.20737 PMC6870902 19235882

[hbm22932-bib-0062] van den Heuvel MP , Sporns O , Collin G , Scheewe T , Mandl RC , Cahn W , Goñi J , Pol HEH , Kahn RS (2013): Abnormal rich club organization and functional brain dynamics in schizophrenia. JAMA Psychiatry 70:783–792. 23739835 10.1001/jamapsychiatry.2013.1328

[hbm22932-bib-0063] Venkataraman A , Whitford TJ , Westin C‐F , Golland P , Kubicki M (2012): Whole brain resting state functional connectivity abnormalities in schizophrenia. Schizophr Res 139:7–12. 22633528 10.1016/j.schres.2012.04.021 PMC3393792

[hbm22932-bib-0064] Wang R , Benner T , Sorensen A , Wedeen V (2007): Diffusion toolkit: A software package for diffusion imaging data processing and tractography. 3720.

[hbm22932-bib-0065] White TP , Joseph V , Francis ST , Liddle PF (2010): Aberrant salience network (bilateral insula and anterior cingulate cortex) connectivity during information processing in schizophrenia. Schizophr Res 123:105–115. 20724114 10.1016/j.schres.2010.07.020

[hbm22932-bib-0066] Whitfield‐Gabrieli S , Thermenos HW , Milanovic S , Tsuang MT , Faraone SV , McCarley RW , Shenton ME , Green AI , Nieto‐Castanon A , LaViolette P (2009): Hyperactivity and hyperconnectivity of the default network in schizophrenia and in first‐degree relatives of persons with schizophrenia. Proc Natl Acad Sci 106:1279–1284. 19164577 10.1073/pnas.0809141106 PMC2633557

[hbm22932-bib-0067] Woods SW (2003): Chlorpromazine equivalent doses for the newer atypical antipsychotics. J Clin Psychiatry 64:663–667. 12823080 10.4088/jcp.v64n0607

[hbm22932-bib-0068] Woodward ND , Karbasforoushan H , Heckers S (2012): Thalamocortical dysconnectivity in schizophrenia. Am J Psychiatry 169:1092–1099. 23032387 10.1176/appi.ajp.2012.12010056 PMC3810300

[hbm22932-bib-0069] Yao Y , Lu WL , Xu B , Li CB , Lin CP , Waxman D , Feng JF (2013): The increase of the functional entropy of the human brain with age. Sci Rep 3:2853. 24103922 10.1038/srep02853 PMC3793229

