# Supplementary material for: Variability of structurally constrained and unconstrained functional connectivity in schizophrenia
Source: Hum Brain Mapp. 2015 Aug 14;36(11):4529–38. doi: 10.1002/hbm.22932 (PMC4843947; doi:10.1002/hbm.22932)
Supplement: Supplementary file 1 — Supporting Information [file HBM-36-4529-s001.docx]

**Supplementary Contents**

[Supplementary material SM1: MRI Acquisition 1](#_Toc421037414)

[Supplementary Material SM2 (and SF1): Resting fMRI Preprocessing 2](#_Toc421037415)

[Supplementary Material SM3: Excluded subjects 4](#_Toc421037416)

[Supplementary Table ST1: Names and abbreviations of AAL brain regions 4](#_Toc421037417)

[Supplementary Figure SF2: Distribution of the structural paths in patients and controls. 5](#_Toc421037418)

[Supplementary Material SM4 (with SF3 and ST3): Anatomical Distribution of Structural Paths 6](#_Toc421037419)

[Supplementary material SM5: Entropy Calculation Method 9](#_Toc421037420)

[Supplementary material SM6: Results with different streamline thresholds (with SF5 and SF6) 11](#_Toc421037421)

# Supplementary material SM1: MRI Acquisition

Diffusion-weighted images were acquired using a single-shot, spin-echo, echo planar imaging (EPI) sequence in alignment with the anterior commissure - posterior commissure (AC-PC) plane. The acquisition parameters were as follows: Repetition Time (TR) = 8.63 s, Echo Time (TE) = 56.9 ms, voxel size = 2mm isotropic, 112 x 112 matrix, Field of View (FoV) = 224 x 224 x 104, flip angle = 90^o^, 52 slices, 32 directions with a b-factor of 1000s/mm2, EPI Factor = 59, total scan time = 6.29 min.

For resting-state fMRI, 240 time points were acquired during the 10 minutes resting phase wherein the subjects were instructed to keep their eyes open and to relax, without the need to focus on any particular task. Dual-echo gradient-echo echo-planar images (GE-EPI) were acquired to enhance sensitivity, using 8-channel SENSE head coil (SENSE factor 2, anterior-posterior direction, TE1/TE2 25/53 ms, flip angle 85°, 255 x 255 mm field of view, in-plane resolution = 3 mm x 3 mm, slice thickness = 4 mm, TR = 2500 ms; 40 descending axial slices, 240 time points per acquisition). Scans were inspected immediately after each acquisition, and if motion was detected, scans were repeated.

A magnetisation prepared rapid acquisition gradient echo image (MPRAGE T1) with 1 mm isotropic resolution, 256 x 256 x160 matrix, TR/TE 8.1/3.7 ms, shot interval 3 s, flip angle 8°, SENSE factor 2 was also acquired for each participant for image registration and to define anatomical regions for tractography.

# Supplementary Material SM2 (and SF1): Resting fMRI Preprocessing

**Correcting for the global signal:** At present, there is no consensus in the field with regard to the removal of global signal when computing functional connectivity related metrics. Global signal removal has been shown to reduce physiological noise from resting fMRI, thus improving its reliability ([Fox, et al., 2009](#_ENREF_3); [Hayasaka, 2013](#_ENREF_5); [Yan, et al., 2013](#_ENREF_12)), though it can increase the frequency of pairwise negative correlation coefficients across the brain ([Saad, et al., 2012](#_ENREF_8)). We employed several procedures to ensure that our results are robust to this issue.

The major argument against the removal of global signal is the introduction of spurious negative correlations; this problem occurs especially when the global signal is of high magnitude and is correlated negatively with a large number of voxels. A data-driven solution for this problem was recently proposed by en et al. ([2012](#_ENREF_2)) who advocate a metric called Global Negative Index (the proportion of all voxels in the brain that show negative correlation with the global signal expressed as a percentage). A GNI value of 3 or greater in a dataset indicates that the removal of global signal using regression will induce a number of spurious negative correlations. For values less than 3, the propensity for spurious anticorrelations will be very low and global signal regression will indeed be advantageous in removing the non-neural sources of the signal. Using this approach we determined the GNI for our sample. The mean GNI was 2.79, indicating that global regression would be advantageous in our dataset. Secondly, in line with Murphy et al. ([2009](#_ENREF_6)), we have refrained from interpreting negative correlation coefficients as representative of anticorrelations. Thirdly, by definition, FCE is a measure that does not depend on the absolute value of the functional connectivity strength. This is illustrated in Figure SF1, on the data obtained from a single random subject. As shown here, while the mean of functional connectivity shifts towards zero upon the removal of global signal, the probability density function shows no change. So, irrespective of whether global signal is retained or removed, FCE values remain the same.

**SF1: Effect of global signal on FCE**


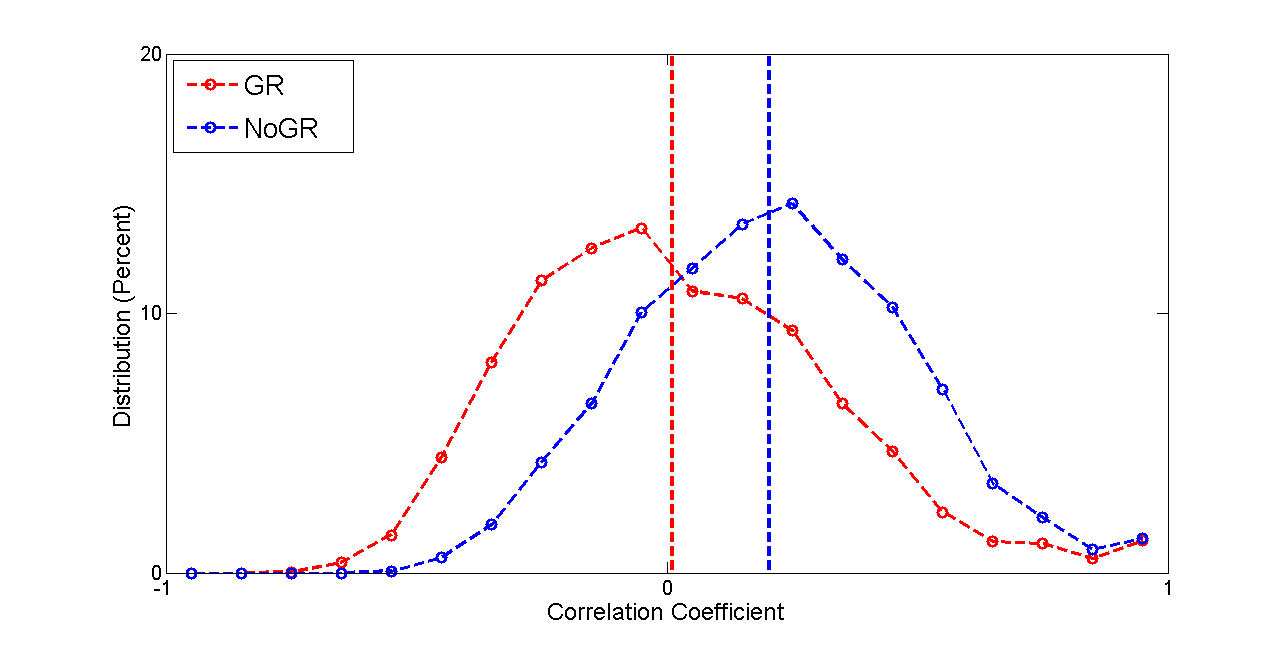


Correlation coefficient distribution for one subject with global signal regression (GR) and without global signal regression (NoGR).

**Correcting for head movements:** In addition to the precautions taken during image acquisition, several other measures were employed to control for movement-induced artefacts. Firstly, motion parameters in three planes were assessed for each participant, and participants with >3 mm or 3^0^ movement were excluded from the analysis. Secondly, displaced frames (defined as frames with summed displacement across all six rigid body motion parameters exceeding 0.5mm or root-mean-square of volume signal intensity difference that exceeded ±3 standard deviation of the average across all scans), along with 1 preceding 2 succeeding frames were replaced using a nearest (unaffected) neighbour interpolation method using ArtRepair software (http://cibsr.stanford.edu/tools/human-brain-project/artrepair-software.html). Thirdly, to remove the variance in functional connectivity spuriously introduced by head motion, we used the head motion parameters as nuisance covariates when extracting the timeseries of BOLD signals. Fourthly, we compared the overall framewise displacement levels between the two groups to detect the possibility of systematic differences in head motion. Finally, we correlated the proportion of displaced frames in each subject with whole brain FCE in each group separately to study the influence of head motion on the group differences in FCE.

No significant difference was noted in the total proportion of frames with displacement (proportion of frames (SD) displaced >0.5mm in controls = 1.2%(0.6%); patients = 6.8%(2%); p=0.13) and for the mean displacement across the 3 translation and 3 rotation axes, quantified in accordance with Power et al. ([2012](#_ENREF_7)) (framewise displacement (SD) in controls = 0.061 (0.29); patients = 0.077(0.54); p=0.14). No significant correlation was noted between mean framewise displacement and FCE of whole brain (r=0.12, p=0.51), primary (r=0.20, p= 0.26) secondary (r=0.07, p=0.68) or tertiary (r=0.13, p=0.46) paths in patients.

# Supplementary Material SM3: Excluded subjects

The original sample consisted of 42 patients and 40 controls, but (3 patients, 5 controls) subjects were excluded due to movement artefacts in fMRI, 1 patient had poor quality DTI due to excessive movement, 2 patients aborted scans (1 DTI, 1 fMRI), and in 2 patients and 3 controls tractography was not successful due to image acquisition errors. There were no differences in the duration of illness (mean (SD) in years in the excluded group=8.9(4.5), included group= 9.6 (8.1), p=0.8), total SSPI score (mean (SD) in the included group= 11.8(7.7), excluded group= 11.6(6.2), p=0.94) or antipsychotic dose (mean (SD) in the excluded group= 694.5 (716), included group= 485.7 (357), p=0.46) between patients who were included or excluded in the analysis.

# ****Supplementary Table ST1: Names and abbreviations of**** AAL brain regions

| **Regions** | **Abbr.** | **RSNs** | **Regions** | **Abbr.** | **RSNs** |
| --- | --- | --- | --- | --- | --- |
| Amygdala | AMYG | 2 | Orbitofrontal cortex (middle) | ORBmid | 3 |
| Angular gyrus | ANG | 1 | Orbitofrontal cortex (superior) | ORBsup | 3 |
| Anterior cingulate gyrus | ACG | 1 | Pallidum | PAL | 2 |
| Calcarine cortex | CAL | 4 | Paracentral lobule | PCL | 6 |
| Caudate | CAU | 2 | Parahippocampal gyrus | PHG | 2 |
| Cuneus | CUN | 4 | Postcentral gyrus | PoCG | 6 |
| Fusiform gyrus | FFG | 4 | Posterior cingulate gyrus | PCG | 1 |
| Heschl gyrus | HES | 5 | Precentral gyrus | PreCG | 6 |
| Hippocampus | HIP | 2 | Precuneus | PCUN | 1 |
| Inferior occipital gyrus | IOG | 4 | Putamen | PUT | 2 |
| Inferior frontal gyrus (opercula) | IFGoperc | 3 | Rectus gyrus | REC | 1 |
| Inferior frontal gyrus (triangular) | IFGtriang | 3 | Rolandic operculum | ROL | 5 |
| Inferior parietal lobule | IPL | 3 | Superior occipital gyrus | SOG | 4 |
| Inferior temporal gyrus | ITG | 2 | Superior frontal gyrus (dorsal) | SFGdor | 1 |
| Insula | INS | 5 | Superior frontal gyrus (medial) | SFGmed | 1 |
| Lingual gyrus | LING | 4 | Superior parietal gyrus | SPG | 3 |
| Middle cingulate gyrus | MCG | 2 | Superior temporal gyrus | STG | 5 |
| Middle occipital gyrus | MOG | 4 | Supplementary motor area | SMA | 6 |
| Middle frontal gyrus | MFG | 3 | Supramarginal gyrus | SMG | 5 |
| Middle temporal gyrus | MTG | 1 | Temporal pole (middle) | TPOmid | 1 |
| Olfactory | OLF | 2 | Temporal pole (superior) | TPOsup | 5 |
| Orbitofrontal cortex (inferior) | ORBinf | 3 | Thalamus | THA | 2 |
| Orbitofrontal cortex (medial) | ORBmed | 1 |  |  |  |

We separated the brain regions on the basis of six Resting State Networks (RSNs) previously reported on the basis of the community structure observed in functional connectivity studies of AAL parcellations ([Guo, et al., 2014](#_ENREF_4); [Tao, et al., 2013](#_ENREF_9)). These networks include RSN1 (Default Mode Network), RSN2 (Subcortical Network), RSN3 (Attention Network), RSN4 (Visual Recognition Network), RSN5 (Auditory Network) and RSN6 (Sensory-motor Network).

# Supplementary Figure SF2: Distribution of the structural paths in patients and controls.


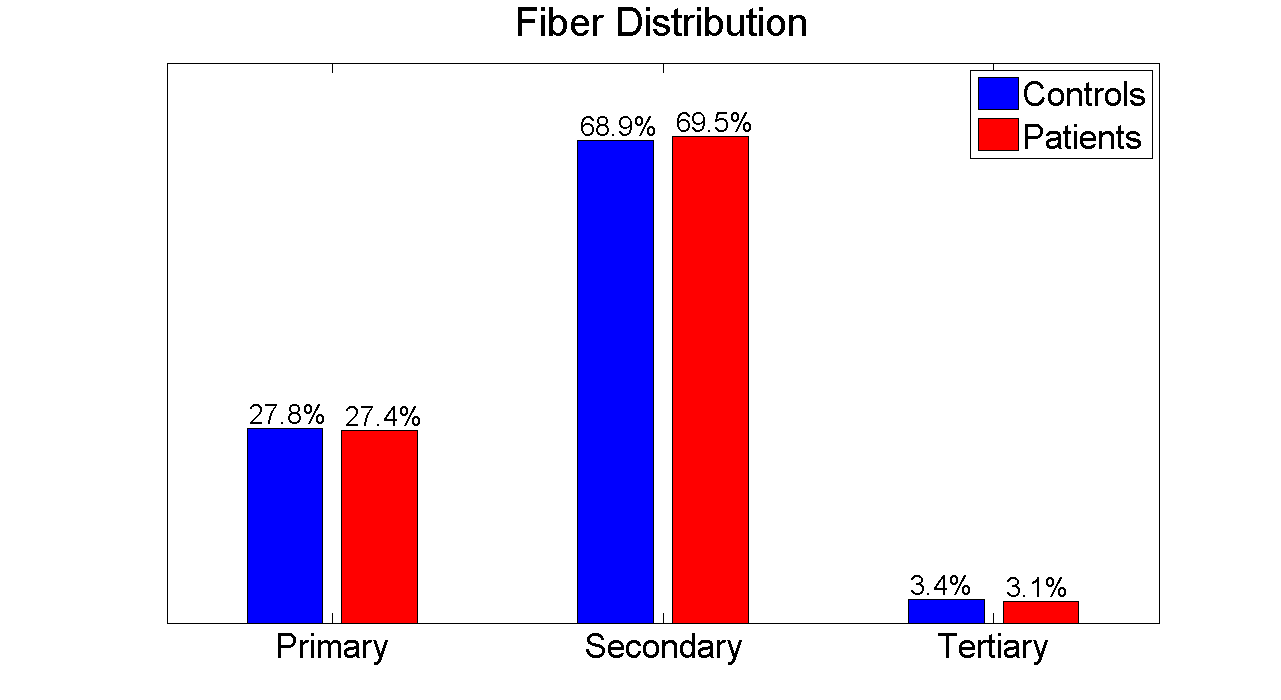


In the main text, we separated primary, secondary and tertiary links based on every individual and calculated corresponding FCE. Thus, when we calculated FCE, the same link could be put into different groups, primary, secondary or tertiary. Figure S2 showed averaged calculations in patients and controls. If a link was considered to be primary in most patients, then it was counted as a primary link. So did secondary and tertiary links.

In total, 1100(27.4%), 2782(69.5%) and 123(3.1%) links were counted as primary, secondary and tertiary ones in patients, while 1113(27.4%), 2757(68.9%) and 135(3.4%) in controls. For patients, on average, links considered as primary were primary in 81.7% subjects, secondary pathways were secondary in 79.8% of subjects and tertiary pathways were tertiary in 68.5% of subjects, while for controls, the respective percentages were 80.8% for primary, 80.4% for secondary and 69.5% for tertiary pathways.

Of the 4005 possible connections, 3774 were in the same category (primary, secondary or tertiary) when comparing patients and controls. A substantial degree of agreement was noted across the three categories of connections when the two groups of subjects were compared. 95.4% of primary, 91.5% of secondary and 75.6% links are classified in the same group in both patients and controls.

# Supplementary Material SM4 (with SF3 and ST3): Anatomical Distribution of Structural Paths

An average correlation matrix in controls and patients, with brain regions organised on the basis of the six Resting State Networks (RSNs) is shown in Figure SF3 below. Table ST2 shows the distribution of the primary secondary and tertiary paths among the links within each RSN (intra-RSN links) and between the RSNs (inter-RSN links). More information about the community structure of the RSNs observed in functional connectivity studies using AAL parcellations can be found elsewhere ([Guo, et al., 2014](#_ENREF_4); [Tao, et al., 2013](#_ENREF_9)).

**Figure SF3. Direct and Indirect structural paths ordered in accordance with the 6 Resting State Networks** in patients (right panel) and in controls (left panel). RSN1 (Default Mode Network), RSN2 (Subcortical Network), RSN3 (Attention Network), RSN4 (Visual Recognition Network), RSN5 (Auditory Network) and RSN6 (Sensory-motor Network).


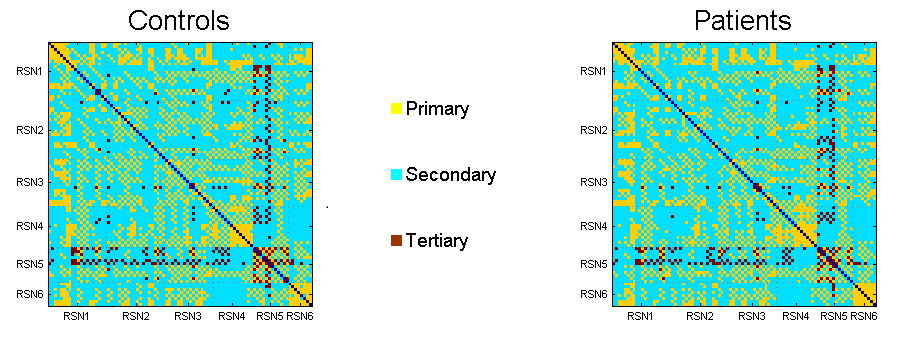


The table below (ST3) show that the RSN1 (Default Mode Network) is the major network with primary structural paths (26%) closely followed by RSN3 (Attention Network). In contrast, for indirect secondary paths, RSN2 (subcortical) is the major substrate (24%). A substantial number of tertiary paths belong to RSN5 (Auditory Network including insula and superior temporal gyrus for >50%).

|  | | **Primary/Secondary/Tertiary**  (Number of inter- and intra-RSN links expressed as a proportion of all primary, secondary or tertiary paths within each group) | | | | | |
| --- | --- | --- | --- | --- | --- | --- | --- |
|  |  | **RSN1** | **RSN2** | **RSN3** | **RSN4** | **RSN5** | **RSN6** |
| **Total** | **Controls** | **26%; 22%; 13%** | **19%; 24%; 16%** | **21%; 17%; 10%** | **16%; 16%; 8%** | **11%; 13%; 51%;** | **10%; 9%; 1%** |
|  | **Patients** | **24%; 22%; 13%** | **19%; 24%; 14%** | **20%; 17%; 12%** | **17%; 16%; 7%** | **11%; 13%; 52%;** | **10%; 9%; 2%;** |
| **RSN 1** | **Controls** | **7%; 4%; 1%** | **5%; 5%; 1%** | **5%; 4%; 1%** | **3%; 4%; 1%** | **2%; 3%; 10%** | **2%; 2%; 0%** |
|  | **Patients** | **7%; 4%; 0%** | **5%; 5%; 0%** | **5%; 4%; 1%** | **3%; 4%; 0%** | **2%; 3%; 12%** | **2%; 2%; 0%** |
| **RSN 2** | **Controls** | **-** | **4%; 5%; 1%** | **4%; 4%; 1%** | **3%; 4%; 1%;** | **1%; 3%; 12%** | **2%; 2%; 0%** |
|  | **Patients** |  | **4%; 5%; 0%;** | **4%; 4%; 1%** | **3%; 4%; 0%** | **2%; 3%; 12%** | **2%; 2%; 0%** |
| **RSN 3** | **Controls** | **-** | **-** | **4%; 3%; 1%** | **3%; 3%; 1%** | **3%; 2%; 6%** | **2%; 1%; 0%;** |
|  | **Patients** |  |  | **4%; 3%; 2%** | **3%; 3%; 1%** | **3%; 2%; 7%** | **2%; 2%; 0%** |
| **RSN 4** | **Controls** | **-** | **-** | **-** | **6%; 1%; 0%** | **1%; 1%; 6%** | **0%; 1%; 0%** |
|  | **Patients** |  |  |  | **6%; 1%; 0%** | **1%; 2%; 5%** | **0%; 2%; 0%** |
| **RSN 5** | **Controls** | **-** | **-** | **-** | **-** | **2%; 1%; 16%** | **1%; 1%; 1%** |
|  | **Patients** |  |  |  |  | **2%; 1%; 14%** | **1%; 1%; 1%** |
| **RSN 6** | **Controls** | **-** | **-** | **-** | **-** | **-** | **2%; 0%; 0%** |
|  | **Patients** |  |  |  |  |  | **3%; 0%; 0%** |

**Table ST3: Distribution of the primary, secondary and tertiary paths across the 6 RSNs**. In the first row, the proportion of all primary, secondary and tertiary paths that involves atleast one brain region from the RSN indicated in the column header has been shown. Subsequent rows show the proportion of inter-RSN and intra-RSN links. Data for controls and patients are displayed separately. The distribution of brain regions within each RSN is shown inn ST1.

# Supplementary material SM5: Entropy Calculation Method

We used entropy to quantify the functioning of the brain. Accordingly, we shall describe this as the functional connectivity entropy (FCE). This part is previously used in the paper Yao, Y., et al. "The increase of the functional entropy of the human brain with age." *Scientific reports* 3 (2013) ([Yao, et al., 2013](#_ENREF_13)){Yao, 2013 #235}.

As outlined below, we view the brain as being divided (parcellated) into a number of distinct regions. For each pair of distinct brain regions, we calculated the correlation coefficient of their neuronal activity; this characterizes the functional coupling of the two brain regions. The resulting set of correlation coefficients generates a frequency distribution. The correlation coefficient of a distinct pair of brain regions, that have been randomly selected, can be regarded as a random variable that follows this frequency distribution. We use the dispersion or variability (measured using Shannon’s entropy, see below) of this random variable as a measure of the FCE (c.f., complexity) of the neuronal dynamics of the brain. We investigate, in this work, how this measure of the FCE changes with age and in Figure SF4 we illustrate the behaviors of the brain’s dynamics that it captures.

Figure SF4 (top row) shows the situation where every brain region fluctuates over time, but is totally correlated with all other regions. In such a case, the FCE of correlation coefficients is zero; all correlation coefficients are unity, and hence their distribution exhibits no randomness, just predictability. A case of non-zero FCE occurs when a range of different correlation coefficients are found between different pairs of brain regions. An example of this case is given by the second row in Figure SF4. In the opposite case of completely independent or incoherent activity in all regions, the correlation coefficients will all be zero and their dispersion (FCE) will again be zero. This means our entropy measure is sensitive to co-ordinated activity that is most interesting, namely activity that is intermediate between fully synchronised and fully incoherent brain-region dynamics.


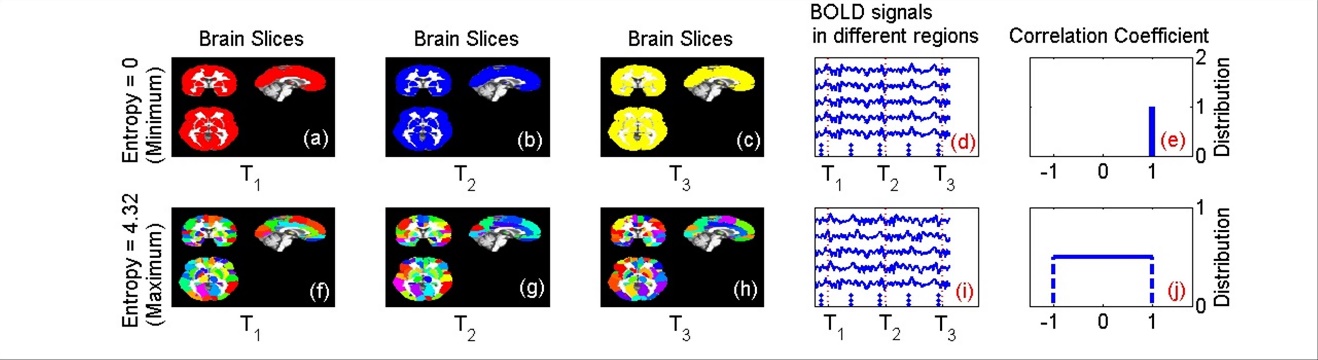


**Figure SF4**

(Figure 1 in Yao, Y., et al. "The increase of the functional entropy of the human brain with age." *Scientific reports* 3 (2013) )

Entropy Calculation Method

After data preprocessing, the time series were extracted in each ROI by averaging the signals of all voxels within the region. The 90 regions were based on a selected atlas, say the AAL Template. After that, we calculated the Pearson Correlation Coefficient of every pair of regions. Since our atlas was the AAL template, we had 4005 function links connecting every two regions. Thus we constructed a whole brain functional network.

Given 4005 different correlation numbers, we required an indicator to represent features of the whole-brain functional network, and considered the values of the correlation coefficients as a realization of a random variable. The range of this was [-1,1]. We then defined the brain functional entropy as the relative entropy, i.e., the KL (Kullback-Leibler) divergence from the correlation distribution to a reference Lebesgue measure. In practice, we did not have a continuous distribution of correlation coefficients, but 4005 correlations values from each individual. We thus separated all 4005 realizations into 20 class intervals of equal width, and determined the frequency (pi) of each class. These frequencies were used to calculate the Shannon entropy (sum of -pi*log(pi)) of the whole brain. This can be considered as the functional connectivity entropy.

# Supplementary material SM6: Results with different streamline thresholds (with SF5 and SF6)

In the primary analysis, we used a minimum threshold of one streamline connecting 2 regions to define a structural path. While the streamline count is often used as a proxy measure of connection in DTI studies, the neurobiological substrate of DTI-based streamlines is unclear. White matter bundles with low FA may have less streamlines than those in the regions with higher FA, despite being equally well connected. Nevertheless, it is possible that a low minimum threshold might have introduced some spurious paths.

We tested the effect of varying the streamline threshold by repeating the primary analysis with a threshold of 2 and a threshold of 3. We found that there were no major changes in the pattern of FCE distribution across the two groups for the different pathways; though the values of statistical significance were altered to some extent, the direction of the results was preserved. These are shown in Figure SF5 and SF6, in line with the Figure 2 in the main text.

It should be emphasized that the use of minimum thresholds is crucial to identify tertiary paths. When a threshold of 10 or more streamlines is used, there would be very few or no tertiary paths that could be identified in the brain. Furthermore, the use of one streamline as a threshold is in line with several DTI-based graph theory applications that generate connection matrices binarised on the basis of having atleast one streamline connection between 2 regions ([Batalle, et al., 2012](#_ENREF_1); [Tymofiyeva, et al., 2013](#_ENREF_10); [van den Heuvel and Sporns, 2011](#_ENREF_11)).


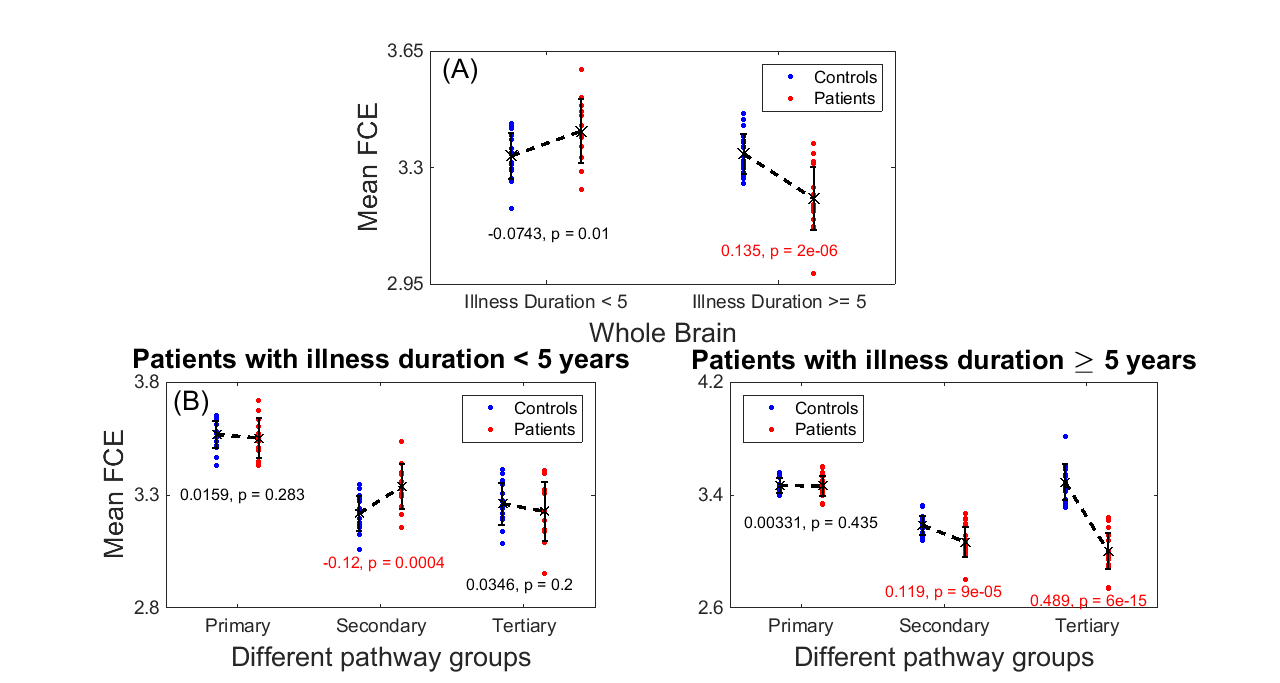


**Figure SF5**

Results of using streamline threshold=2


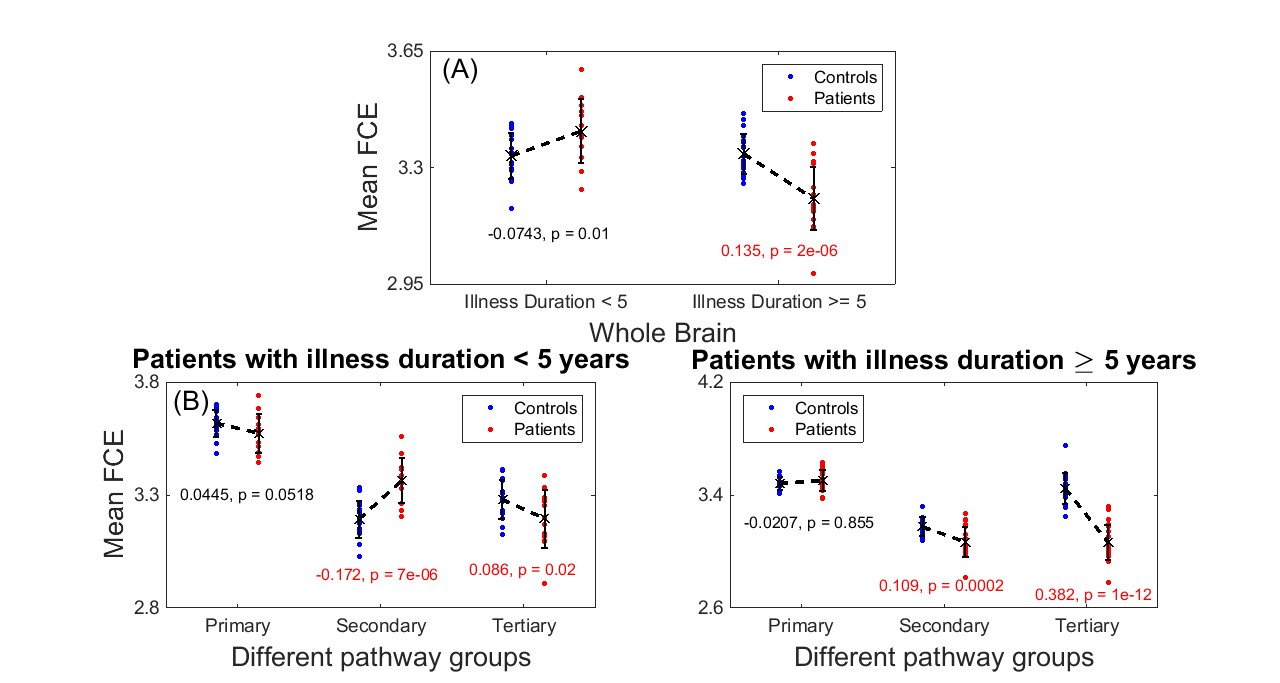


**Figure SF6**

Results of using streamline threshold=3

**References**

Batalle, D., Eixarch, E., Figueras, F., Muñoz-Moreno, E., Bargallo, N., Illa, M., Acosta-Rojas, R., Amat-Roldan, I., Gratacos, E. (2012) Altered small-world topology of structural brain networks in infants with intrauterine growth restriction and its association with later neurodevelopmental outcome. Neuroimage, 60:1352-1366.

Chen, G., Chen, G., Xie, C., Ward, B.D., Li, W., Antuono, P., Li, S.J. (2012) A method to determine the necessity for global signal regression in resting‐state fMRI studies. Magnetic Resonance in Medicine, 68:1828-1835.

Fox, M.D., Zhang, D., Snyder, A.Z., Raichle, M.E. (2009) The global signal and observed anticorrelated resting state brain networks. Journal of neurophysiology, 101:3270-3283.

Guo, S., Kendrick, K.M., Yu, R., Wang, H.L., Feng, J. (2014) Key functional circuitry altered in schizophrenia involves parietal regions associated with sense of self. Hum Brain Mapp, 35:123-39.

Hayasaka, S. (2013) Functional connectivity networks with and without global signal correction. Frontiers in human neuroscience, 7.

Murphy, K., Birn, R.M., Handwerker, D.A., Jones, T.B., Bandettini, P.A. (2009) The impact of global signal regression on resting state correlations: are anti-correlated networks introduced? Neuroimage, 44:893-905.

Power, J.D., Barnes, K.A., Snyder, A.Z., Schlaggar, B.L., Petersen, S.E. (2012) Spurious but systematic correlations in functional connectivity MRI networks arise from subject motion. Neuroimage, 59:2142-2154.

Saad, Z.S., Gotts, S.J., Murphy, K., Chen, G., Jo, H.J., Martin, A., Cox, R.W. (2012) Trouble at rest: how correlation patterns and group differences become distorted after global signal regression. Brain connectivity, 2:25-32.

Tao, H., Guo, S., Ge, T., Kendrick, K.M., Xue, Z., Liu, Z., Feng, J. (2013) Depression uncouples brain hate circuit. Mol Psychiatry, 18:101-11.

Tymofiyeva, O., Hess, C.P., Ziv, E., Lee, P.N., Glass, H.C., Ferriero, D.M., Barkovich, A.J., Xu, D. (2013) A DTI-based template-free cortical connectome study of brain maturation.

van den Heuvel, M.P., Sporns, O. (2011) Rich-club organization of the human connectome. The Journal of neuroscience, 31:15775-15786.

Yan, C.-G., Cheung, B., Kelly, C., Colcombe, S., Craddock, R.C., Di Martino, A., Li, Q., Zuo, X.-N., Castellanos, F.X., Milham, M.P. (2013) A comprehensive assessment of regional variation in the impact of head micromovements on functional connectomics. Neuroimage, 76:183-201.

Yao, Y., Lu, W.L., Xu, B., Li, C.B., Lin, C.P., Waxman, D., Feng, J.F. (2013) The Increase of the Functional Entropy of the Human Brain with Age. SCIENTIFIC REPORTS, 3:2853.
