# Supplementary material for: Variability of structurally constrained and unconstrained functional connectivity in schizophrenia
Source: Hum Brain Mapp. 2015 Aug 14;36(11):4529–38. doi: 10.1002/hbm.22932 (PMC4843947; doi:10.1002/hbm.22932)
Supplement: Supplementary file 2 — Supporting Information [file HBM-36-4529-s002.docx]

Reviewer: 1

**Comments to the Author**

**The authors have adequately addressed most of the reviewers' concerns, and the paper has improved. The paper is valuable in its combination of functional and anatomical connectomics, which I think is indeed a very important and largely unaddressed issue.**

**However, I suggest 2 final edits because I think that the responses have**

**been incomplete in places: - The answer to the question of inter-individual variability in network structures is quite clear. However it does not specify the overlap between patients and controls in terms of each type of connection. The current information implies that, especially for tertiary networks, there may be marked differences between individuals and diagnostic groups in the anatomical regions that are involved. In total there are >200 mismatches in the classifications of the connections, and there are only ~130 tertiary connections. Thus, an alternative interpretation for some of the results is that it is not the functional connectivity between regions that is altered, but their classification. This alternative explanation should either be explored or added to the discussion section.**

We thank the reviewer for a favourable opinion and highlighting this point again. We now present the results of the group overlap for each type of connections separately, and to highlight the importance of this, included these results in the main text, with a note added in the discussion section.

*Information in the results section of the manuscript: Of the 4005 possible connections, 3774 were in the same category (primary, secondary or tertiary) when comparing patients and controls. A substantial degree of agreement was noted across the three categories of connections when the two groups of subjects were compared. 95.4% of primary, 91.5% of secondary and 75.6% links are classified in the same group in both patients and controls.*

*Text added to discussion section: Our results also suggest that while there is a substantial overlap in the anatomical similarity of primary and secondary paths between patients and controls, 24.4% of all identified tertiary paths were present only in one group of subjects, highlighting that the weaker, indirect links are more prone to spatial redistribution, in addition to changes in the strength of connectivity.*

**- I agreed with reviewer 1 (comment 1), regarding some over-interpretation**

**of the results. I do not see how the authors have addressed this main**

**concern (e.g. the main final conclusion remains unaltered). As far as I**

**understand, increased entropy, as in the metric used here, means just as much increased randomness as increased information. The reference to Gestalt theory (of the whole and its parts) does not make sense in my opinion.**

In general, the entropy of a variable is the ‘amount of information’ that it contains. This ‘amount’ relates to the relative ease with which one can guess the value of a variable without having to look at the raw data: the easier it is to guess the value of the variable, the more predictable it becomes, and the amount of information that it contains could be deemed low. In other words, the state of a low entropy variable provides limited information about the space in which it is embedded. This inference was elegantly captured by Tononi et al., by invoking the Gestalt philosophy: Higher entropy within a system suggests that the whole provides more information than the sum of its parts (Tononi et al., 1994).

We agree that invoking Tononi et al., may reduce the clarity of our inference. So we have now modified this statement as below:

*To conclude, in a healthy brain, the resting connectional architecture (system as a whole) accommodates a higher degree of variability in functional connections (constituent parts), but in established schizophrenia the variability of constituents that make up this system is limited, especially when considering those with an established illness.*

**Reviewer: 2**

**Comments to the Author**

**The authors have thoroughly answers to the issues I raised. I think that the manuscript is now well suited for publication.**

We thank the reviewer for a favourable opinion.
